# Bacterial leakage assessment in root canals sealed with AH Plus sealer modified with silver nanoparticles

**DOI:** 10.1186/s12903-021-01924-2

**Published:** 2021-11-12

**Authors:** Farzaneh Afkhami, Shifteh Nasri, Sara Valizadeh

**Affiliations:** 1 Department of Endodontics, School of Dentistry, Tehran University of Medical Sciences, Tehran, Iran; 2 Tehran University of Medical Sciences, Tehran, Iran; 3 Restorative Dentistry Department, School of Dentistry, Tehran University of Medical Sciences, Tehran, Iran

**Keywords:** Bacterial leakage, Canals sealer, Endodontics, Metal nanoparticles, Silver nanoparticles, Root canal therapy

## Abstract

**Background:**

Successful endodontic therapy requires prevention of bacterial leakage between the root canal filing and root-canal walls. Sealing quality of a root canal filling depends strongly on the sealing ability of the sealer used. The present study aimed to evaluate the bacterial leakage resistance of AH Plus sealer modified with silver nanoparticles.

**Methods:**

Forty sound teeth were obturated using lateral compaction technique except for five teeth as the negative controls. After considering five teeth as the positive controls (filled without sealer), the remaining teeth were assigned to two groups (n = 15) in terms of the sealer used (AH Plus sealer and silver nanoparticle-modified AH Plus). Bacterial leakage was evaluated in saliva using the two-chamber technique in every 24 h. When all the samples in the test groups were contaminated, the study was terminated. The data were analyzed with log-rank statistical test.

**Results:**

All samples in both experimental groups were contaminated during the 3-months period of observation. There was no significant difference in contamination time between study groups (*P* > 0.05).

**Conclusions:**

Silver nanoparticles used in tested concentration did not improve the bacterial leakage resistance of AH Plus sealer.

## Background

A challenge in endodontic treatment is to reduce bacterial biofilms in the root canal, followed by the root canal’s proper sealing to provide the opportunity for the host to repair and regenerate injured tissues [1]. Thorough eradication of microorganisms from the root canal system is necessary to ensure treatment success; however, it appears that the bacteria persist in the root canal after root canal treatment [2]. The main aim of root canal treatment is to significantly eradicate the microorganisms, and to modify the conditions in the root canal system less favorable for microbial reentry and regrowth. Despite attempts to disinfect the root canal system, treatment failure and re-contamination of the root canal system occur due to the complexity of the root canal system and the resistant nature of biofilms [3–6].

Failure in eliminating bacteria from the root canal system during endodontic treatment has been considered an essential factor for periapical inflammation, destruction of periapical tissues, periapical lesions, and endodontic treatment failure [7]. According to previous studies, 79% of root canals with residual bacteria exhibited evidence of the healing of periapical lesions [1].

Although the main obturating material for root canals is gutta-percha, it cannot alone fill the entire root canal space and adhere to its walls. Therefore, endodontic sealers have a vital role in sealing the three-dimensional system of the root canal in association with gutta-percha [8]. Unfortunately, no material currently exists that can alone, or in combination with other materials, provide a thorough seal for the root canal space, and sub-micron spaces always remain between the root canal walls and the obturating materials. Clinically, sealers with antibacterial activity can help decreasing the residual bacteria and prevent the re-contamination of the root canal space with microorganisms after successful endodontic treatment [9].

Endodontic sealers can fill the irregularities between the root canal wall and gutta-percha; they can also adhere to the root canal wall and penetrate the dentinal tubules. Most sealers have antibacterial activity [10]. However, efforts are underway to incorporate antibacterial agents into sealers to prolong or increase their antibacterial activity. Nonetheless, most of these materials depend on the release of the agents in the environment, which are released and neutralized over time [11].

Nanotechnology is a new approach in all the health-related fields, including dentistry. Disinfecting the root canals with nanoparticles has become very popular in recent years [12]. Silver nanoparticles are one of the most common antibacterial agents that are incorporated into composite resins, bonding agents, and materials used during endodontic treatment [13]. Silver nanoparticles have been incorporated into endodontic sealers and medicaments in recent studies, which results in higher antibacterial properties at low concentrations, with no adverse effects on the mechanical properties as well as teeth discoloration [14–16].

Silver nanoparticles’ mechanism of action is through the release of silver ions that target separate points on the bacteria. For example, silver ions physically adhere to the bacterial cell membrane, increase its permeability, and affect the movement of essential ions into and out of the bacterial cell [16]. Besides, these nanoparticles destroy the bacterial wall, releasing the cellular components. The silver ions bind to the sulfhydryl groups in proteins due to their high affinity for sulfur, nitrogen, and oxygen, denaturing proteins; also, they bind to the nitrogen atoms in nuclear acids and prevent DNA replication [17, 18].

Therefore, the present study aimed to evaluate bacterial leakage using conventional AH Plus sealer and AH Plus sealer modified with silver nanoparticles. The null hypothesis was there is no difference in bacterial leakage between these two sealers.

## Methods

The study protocol was approved by the Ethics Committee of Tehran University of Medical Sciences (IR.TUMS.1394/1075).

Forty sound human single-canal teeth extracted for orthodontic or periodontal reasons were included in the present in vitro study. Before extraction, patients (all of them were above 18) were informed and signed the informed consent. All the teeth had matured closed apices, were caries-free, and had no cracks, resorption, and calcification. Soft tissues and calculi were removed from the tooth surfaces with scaling curettes. The presence of only one root canal was confirmed by parallel radiography in both mesiodistal and buccolingual directions. The absence of any cracks in the root was evaluated before the study procedures with a dental loupe (Reister, Jungingen, Germany).

### Sample preparation

A highspeed fissure bur (Dentsply International, York, PA) was used to remove the crowns at a right angle to the tooth long axis under air and water spray to achieve a standard root length of 16 mm in all the samples. The working length (WL) was determined 1 mm short of the length at which the tip of a #15K-file (Dentsply Maillefer, Ballaigues, Switzerland) was visible at the apical foramen.

The coronal third was prepared with #3, #2, and #1 Gates-Glidden drills (Dentsply International Inc, Pennsylvania, USA). Then the S1, S2, F1, F2, F3, F4, and F5 files of the ProTaper rotary system were used, respectively, to prepare the root length up to the WL. Root canal irrigation during instrumentation were carried out with 1 ml of 2.5% NaOCl after each file. RC-Prep (Premier, North America, United States) was used as a lubricant during instrumentation. One operator carried out all the preparation steps. At the end of root canal preparation procedures, 3 ml of 17% EDTA (ethylenediaminetetraacetic acid) was used to remove the smear layer and followed by 3 ml of 5.25% NaOCl solution each for 3 min. Finally, the root canals were irrigated with 5 ml of normal saline and dried with paper points.

Two layers of nail varnish were applied to all the root surfaces except for the root canal orifice and 2 mm of the root end in all the groups except the negative control group to prevent microbial contamination through the external surfaces of the samples and the dentinal tubules into the primary and accessory root canals. In the negative control group, all the root surfaces were coated with nail varnish to seal the samples thoroughly. The samples were then autoclaved for sterilization. Therefore, all the remaining procedures were carried out in sterile conditions under a hood.

Five samples were filled without sealer and assigned to the positive control group. The remaining teeth were assigned to two experimental groups (n = 15) based on the sealer used. In AH Plus group, AH Plus sealer (Dentsply DeTrey, Konstanz, Germany), and in the AH Plus/silver nanoparticles group, AH Plus sealer modified with 200 ppm of silver nanoparticles were used. The nanoparticles measured 20 nm at average with 1:1 volume and were mixed with the sealer by vortexing. The composition of the AH Plus sealer is presented in Table 1.

**Table 1:** The composition of AH Plus sealer

AH Plus	Dentsply DeTrey, Konstanz, Germany	Epoxy paste: diepoxy, calcium tungstate, zirconium oxide, aerosol, dye Amine paste: 1-adamantane amine, N. N′dibenzyl-5 oxanonandiamine-1,9, TCD-diamine, calcium tungstate, zirconium oxide, aerosol, and silicone oil

Root canals were dried with paper points #40 (Tanari, Bologna, Italy). Sealers were applied inside the root canals with lentulo spiral. Gutta-percha (Meta Biomed Co. Ltd, Cheongwon Korea) was used to obturate the root canals using the lateral compaction technique. The samples in the negative control group were not obturated.

A heat carrier was used to remove 2 mm of gutta-percha from the coronal third of the obturation. The extra sealer was eliminated from the root canal orifice walls with a cotton pellet impregnated with alcohol.

At this stage, no material was used as an orifice plug or for coronal sealing so that the antibacterial activity of the sealers could be evaluated. The samples were coded and placed in special packs, followed by incubation at 100% relative humidity at 37 °C for 24 h to allow setting of the sealers.

### Bacterial leakage evaluation

**Fig. 1:**
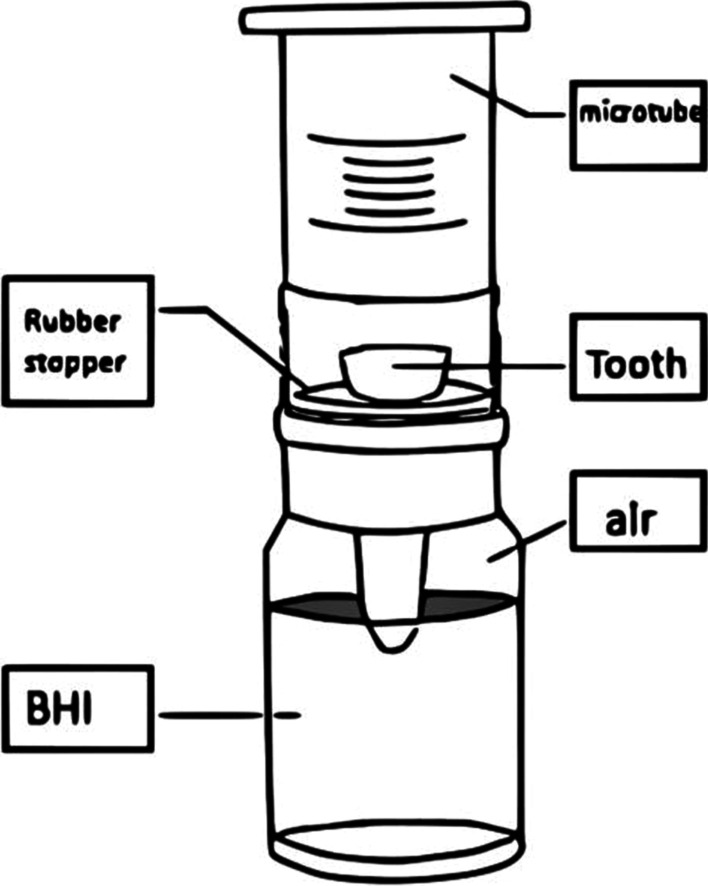
The two-chamber model for testing bacterial leakage

A two-chamber model (Fig. 1) similar to that in the study by Fathi et al. [18] was used to evaluate coronal microleakage in the present study. The coronal 2-mm of the root was placed in a cut-ended microtube, and the rest of the root length was placed out of it.

Then an acrylic resin bur (Dentsply International, York, PA) in a handpiece was used in the laboratory to create a hole in the cap of penicillin vial, and the root was placed through the cap of penicillin vial. Then two layers of glue (UHU, Bühl, Germany) were applied in all the samples to seal the tooth–microtube and microtube–rubber cup interfaces to prevent bacterial leakage in the test environment.

The prepared samples consisting of teeth, microtubes, and the penicillin vial caps were sterilized with ethylene oxide gas for 12 h. Then sterile brain heart infusion (BHI) broth was added to the vials so that the 2 mm of the samples’ root ends were immersed in sterile BHI. The procedure was carried out under a laboratory hood in a sterile environment. The surfaces of all the prepared samples were covered with glue to seal all the interfaces. Then the samples were incubated at 100% relative humidity at 37 °C for 5 days without adding saliva. The lack of turbidity indicated the absence of contamination and the samples’ sterility. After ensuring the samples’ sterility, natural human saliva was added once every 3 days to the samples’ environment (the space within the microtube). The samples were evaluated every day for turbidity, which continued until turbidity was detected on all the samples in the BHI environment. Microbial contamination leakage was indicated by the turbidity of the liquid culture environment. The samples were evaluated for 120 days, similar to the study by Fathi et al. [19]. The day that turbidity was seen in each sample was recorded and the turbid samples were excluded.

The microbial cultures were carried out from the BHI culture environment to identify the bacterial species with the highest ability to penetrate the root canal, causing turbidity in the BHI culture medium.

### Evaluation of bacterial culture

A graduated pipette was used to passage one drop (0.05 ml) of the turbid culture medium to the blood agar, eosin methylene blue agar, and ENB agar culture media.

A sterile needle was then used to culture some of the colonies formed on the differential TSI (triple sugar iron), SIM (sulfide indole motility), citrate, and urea specific culture media to determine and diagnose the bacterial species. The plates were incubated for 24 h after culturing all the samples.

### Statistical analysis

The log-rank test was used to compare the leakage time between the different study groups at a statistical significance level of *P* < 0.05.

## Results

Evaluation of bacterial microleakage after 120 days showed no contamination and turbidity in the negative control group, indicating the sterility and lack of contamination of the test environment. Turbidity was observed in all the positive control group samples.

Based on statistical analyses, there were no significant differences in the microleakage time between the study groups (*P* > 0.05). Table 2 presents the means and standard deviations of microleakage days.

**Table 2:** The means and standard deviations of microleakage days in the study groups

Group	Minimum (d)	Maximum (d)	Means ± standard deviations (d)
AH Plus	5	91	39.80 ± 27.97
AH Plus/silver nanoparticles	6	97	37.13 ± 20.21

The results showed that all the samples in both study groups exhibited bacterial leakage after almost 1–3 months, indicating that none of the sealers could prevent bacterial leakage in the study groups.

In the AH Plus group, 40% of the samples (n = 6), 53% of the samples (n = 8), and 7% of the samples (one sample) were contaminated up to day 30, between days 30 and 60, and up to day 91, respectively. In the AH Plus/silver nanoparticles group, 46.6% of the samples (n = 7), 33.3% of the samples (n = 5), and 20.1% of the samples (n = 3) were contaminated up to day 30, between days 30 and 60, and up to day 97, respectively.

The differential table of the culture media after evaluating the samples for 24 h and isolation of bacteria showed that the bacteria with the highest penetration ability in both sealer groups were *Klebsiella* and *E. coli*.

## Discussion

The results of this study confirmed the null hypothesis and showed no significant difference in bacterial leakage resistance between two tested endodontic sealers.

The elimination of bacteria and microorganisms from the three-dimensional root canal space is not possible with root canal preparation and obturation only. Endodontic sealers with antimicrobial activity can destroy residual microorganisms in the root canals [20]. Therefore, the present study aimed to evaluate the effect of two sealers on preventing bacterial leakage. The bacterial microleakage method was used in the present study to evaluate leakage. This method has extensively been evaluated and used in different studies and is more biological and clinical than other methods. Moreover, this model most closely simulates clinical situation [21, 22].

The results of studies using dyes or electrochemical isotopes in some laboratory models to evaluate bacterial leakage might be different from reality due to their smaller molecular size than the real diffusion. Besides, the presence of voids and prevention of dye penetration might jeopardize the results of studies designed on this basis. A study by Oliver and Abbot showed that the dye penetration method to evaluate the leakage of root canal obturation materials is associated with low validity [23].

Some studies have used *Streptococcus salivarius* [24] and obligate anaerobic bacteria [25] to evaluate microleakage. The use of different bacterial species alone does not correspond to the clinical conditions because the penetration of saliva into the root canal(s) of endosmotically treated teeth occurs in the oral environment. Many bacterial species have reciprocal nutritional and commensal interactions with each other in the saliva (4). Tselnic et al. [26] showed that the use of natural saliva to evaluate bacterial microleakage in endodontically treated teeth yields results that are real and close to the clinical condition. Therefore, natural human saliva was used in the present study.

Achieving a proper seal is one of the main objectives of root canal treatment, and several materials can achieve such a seal. AH Plus sealer was evaluated in the present study because it exhibits better adhesion to dentin due to covalent bonds with the dentin collagen [27].

A systematic review showed that sealers have a critical role in preventing microleakage into the root canal system. The most commonly used sealers are resin sealers, eugenol-based sealers, and calcium hydroxide-based sealers. Although these sealers exhibit antimicrobial activity when they are freshly mixed, they lose this property over time partially or completely [28]. Another review study showed that the antimicrobial activity of sealers persists for a maximum of 1 week. Therefore, the incorporation of materials with antimicrobial properties into sealer has always been considered. Nanoparticles are added to sealers to decrease bacterial penetration, increase antimicrobial activity within the dentinal tubules, and increase sealers’ strength [29].

Besides, most sealers have antibacterial and cytotoxic effects, which might limit bacterial invasion. The incorporation of antibiotics and/or silver nanoparticles has been suggested to increase sealers’ antibacterial activity [30]. Silver nanoparticles have antibacterial and antifungal activities and disrupt bacterial membrane integrity by penetrating these membranes and increasing their permeability. These nanoparticles exhibit proper action against oral pathogens [31].

In the present study, orifice plugs or coronal seal was not used to only evaluate the antimicrobial activity of the obturating material in association with AH Plus sealer and AH Plus sealer modified with silver nanoparticles. In both groups, all the samples exhibited bacterial contamination after 3 months, i.e., none of the sealers could prevent bacterial leakage for more than 3 months.

Re-contamination of the root canal system occurs after the dissolution of the sealer in the saliva at the sealer–root canal wall or sealer–gutta-percha interface. Bacteria and their by-products and other salivary irritants can penetrate through the apical foramen and accessory canals into the periradicular tissues due to the absence of a coronal seal [32].

Kangarlou et al. [33] reported that the incorporation of silver nanoparticles into the AH Plus sealer did not significantly increase antibacterial activity. Another study showed that it was safe to add silver nanoparticles, and they preserved their antimicrobial activity over time [17]. Silver nanoparticles are water-soluble and can be dissolved in organic solvents; they have large contact surfaces due to small particle size; therefore, they exhibit high biocompatibility. Besides, when they are mixed with other materials, their properties are not adversely affected.

AH Plus is an epoxy resin-based sealer, which cannot prevent bacterial leakage alone or in combination with silver nanoparticles. Zhang et al. showed that the AH Plus sealer in the modified direct contact test preserved its antimicrobial activity for 1 day, and the freshly mixed sealer exhibited higher antimicrobial activity, consistent with the present study. However, on the first day of the experiment, microbial contamination did not occur, and bacterial leakage occurred after the fifth day [34].

In the present study, silver nanoparticles were added to the AH Plus sealer to produce a sealer with antimicrobial properties. Since the root canal structure has many irregularities, and some of its areas might not be affected by the available instruments, a sealer with antibacterial activity that can flow to penetrate the complex anatomy of the root canal is optimal; however, it should not be excessively flowable to extrude into periapical tissues [35]. According to ISO6876/2012, endodontic sealers should have at least 20 mm of flow [36]. The incorporation of silver nanoparticles improves the flow of the AH Plus sealer [37].

In a study by Baras et al. [38], the incorporation of 0.15% silver nanoparticles and DMAHDM (dimethylaminohexadecyl methacrylate) increased the antimicrobial activity of the sealer with no adverse effects on the sealer’s other properties. Besides, Seung et al., showed that the incorporation of 2.5% quaternary ammonium DMAHDM and 0.15% silver nanoparticles did not affect the physical properties of AH Plus sealer, such as flow, setting time, dimensional changes, and solubility. However, this sealer exhibited significantly higher antibacterial activity against *E. faecalis* 14 days after setting [39]. The discrepancies between these studies and the present study might be attributed to differences in the concentration of nanoparticles or the presence of DMAHDM.

Teixira et al. evaluated the antibacterial effect and physical properties of different sealers after adding silver nanoparticles (AgVO3) at 0%, 2.5%, 5%, and 10% concentrations. DCT (direct contact test) after 48 h and 7 days showed that the incorporation of silver nanoparticles significantly increased the antibacterial activity of the sealers, with no change in their physical properties [40]. The differences in the results of that study and the present study might be explained by differences in the silver nanoparticles used, their concentrations, and the evaluation methods. In addition, another study showed that the incorporation of silver nanoparticles (AgVO3) at 0%, 2.5%, and 10% concentrations into AH Plus sealer did not significantly affect the antibacterial activity of the freshly mixed sealer and 30 days after mixing with the DCT method against *E. faecalis* [37], consistent with the present study.

According to the results of various previous studies, it is possible to consider the antibacterial effects of different nanoparticles, including silver nanoparticles, as a strategy to eliminate bacterial biofilms from the root canals. With advances in nanoparticle technology, it is possible to use nanoparticles to improve root canal disinfection and seal the root canal space during root canal treatment. However, data on the treatment efficacy and their release should be adequately understood for effective treatment with these nanoparticles [41].

Despite the importance of in vitro studies, extending the results of these studies to the clinical setting should be carried out with caution because confounding factors related to patients and dentists can be controlled with some difficulty. Some studies have shown that the smear layer prevents the penetration of sealers into the dentinal tubules. Therefore, the root canal system can affect the antibacterial activity of different materials [42].

## Conclusion

Under the conditions of the present study, it can be concluded that the incorporation of silver nanoparticles into the AH Plus sealer cannot improve its bacterial leakage resistance property, necessitating proper coronal sealing after endodontic treatment.

## Data Availability

The datasets used during the current study are available from the corresponding author on reasonable request. All data analyzed during this study are included in this published article in the form of tables and figures.
